# Supplementary material for: Negative regulation of type I interferon signaling by integrin-linked kinase permits dengue virus replication
Source: PLoS Pathog. 2023 Mar 17;19(3):e1011241. doi: 10.1371/journal.ppat.1011241 (PMC10057834; doi:10.1371/journal.ppat.1011241)
Supplement: S1 Text — (DOCX) [file ppat.1011241.s001.docx]

**Supporting information of**

**Negative regulation of type I interferon signaling by integrin-linked kinase permits dengue virus replication**

Yi-Sheng Kao, Li-Chiu Wang, Po-Chun Chang, Heng-Ming Lin, Yee-Shin Lin, Chia-Yi Yu, Chien-Chin Chen, Chiou-Feng Lin, Trai-Ming Yeh, Shu-Wen Wan, Jen-Ren Wang, Tzong-Shiann Ho, Chien-Chou Chu, Bo-Cheng Zhang and Chih-Peng Chang.

**Materials and Methods**

**Establish the U937 cells with ILK knockdown**

The human monocytic cell line U937 was maintained in the medium according to the instructions of the American Type Culture Collection. The U937 cells were infected with lentiviruses carrying control (shLuc) or shRNA targeting 3’UTR of ILK (shILK) in medium with 8 mg/ml polybrene for 48 hours and selected in medium containing puromycin.

**Inactivation of DENV by ultraviolet (UV) light**

To prepare the UV-inactivated virus, DENV was exposed to a UVGL-58 Handheld UV lamp at wavelength 254 nm for 20 minutes. The loss of infectivity in DENV was confirmed by plaque assays.

**Western blot analysis**

Lysates of cells mock-infected or infected with DENV or UV-inactivated DENV or of cells treated with recombinant IFN-β (PERPROTECH) were subjected to western blot analysis using primary antibodies against NS1, NS3, NS4B, ILK, SOCS3, p-STAT1, STAT1, p-STAT2, STAT2, LC3 (MBL International), caspase-3 (Cell Signaling Technology), and β-actin (Abcam) and HRP-conjugated secondary antibodies. Protein bands were detected using an enhanced chemiluminescence substrate kit (PerkinElmer). The intensity of protein bands was measured using ImageJ software.

**Flow cytometry**

Cells were infected with DENV (MOI=25) at 4°C for 2 hours, harvested, and stained with 1 μg/ml anti-E antibody (clone 137-22) for 1 hour and Alexa 488-conjugated secondary antibody (goat anti-mouse IgG) for another 1 hour on ice. The binding of DENV to cells was determined and analyzed by a Flow Cytometer (BD).

**RT-PCR**

Total RNA was prepared via a RNeasy RNA Mini Kit (Qiagen) following the manufacturer’s instructions. The cDNA was reverse transcribed from 1 μg total RNA by random primers. For RT-PCR, equal amounts of cDNA were subjected to PCR reactions with primers as follows: IFN-α forward CTTGATGCTCCTGGCACAGA and reverse TCATGGAGGACAGGGATGGT; IFN-β forward TAGCACTGGCTGGAATGAGA and reverse TCCTTGGCCTTCAGGTAATG; IFN-λ forward CGCCTTGGAAGAGTCACTCA and reverse GAAGCCTCAGGTCCCAATTC; SOCS1 forward TTGCCTGGAACCATGTGG and reverse GGTCCTGGCCTCCAGATACAG; SOCS3 forward GGAGTTCCTGGACCAGTACG and reverse TTCTTGTGCTTGTGCCATGT; GAPDH forward CCACTCCTCCACCTTTGACG and reverse CCACCACCCTGTTGCTGTAG. ATP6V0C forward GAATGACGACATCAG CCTCTACA and reverse GATCATGCCCACGAATAGTCGG. PCR products were separated in 2% agarose DNA gel.

**Proximity ligation assay**

Mock infected or DENV infected cells were fixed with cold 4% paraformaldehyde and stained with anti- ILK, -NS1, -NS3 or -NS5 antibodies to determine the detecting protein-protein interactions by Proximity Ligation Assay kit (Sigma-Aldrich) according to the manufacturer’s instructions.
