# Supplementary figures and images for: Negative regulation of type I interferon signaling by integrin-linked kinase permits dengue virus replication
Source: PLoS Pathog. 2023 Mar 17;19(3):e1011241. doi: 10.1371/journal.ppat.1011241 (PMC10057834; doi:10.1371/journal.ppat.1011241)

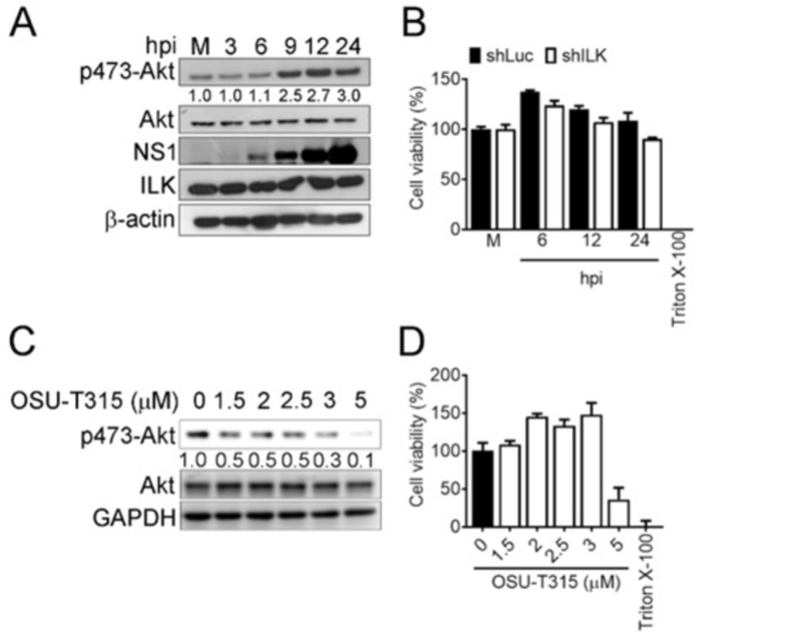

Supplement: S1 Fig — (A) Representative western blots of phosphorylated (p-) Akt at Ser473 and indicated proteins in mock- (M) and DENV-infected A549 cells at indicated hours post-infection (hpi) are shown. The ratios of phosphorylated to total Akt relative to mock-infected cells are shown. (B) The viability of control (shLuc) and ILK knockdown (shILK) A549 cells with mock-infected, infected with DENV at indicated hpi, or treating with 0.05% Triton X-100 for 24 hours are shown. The viability of mock-infected control cells was set as 100%. (C and D) Representative western blots of indicated proteins (C) and cell viability (D) of A549 cells treated with indicated concentration of OSU-T315 for 24 hours are shown. The ratios of phosphorylated to total Akt relative to cells treated with 0 μM OSU-T315 are shown. The viability of cells treated with 0 μM was set as 100%. Data represent means + SD (error bars) in bar graphs. (TIF) [file ppat.1011241.s002.tif]

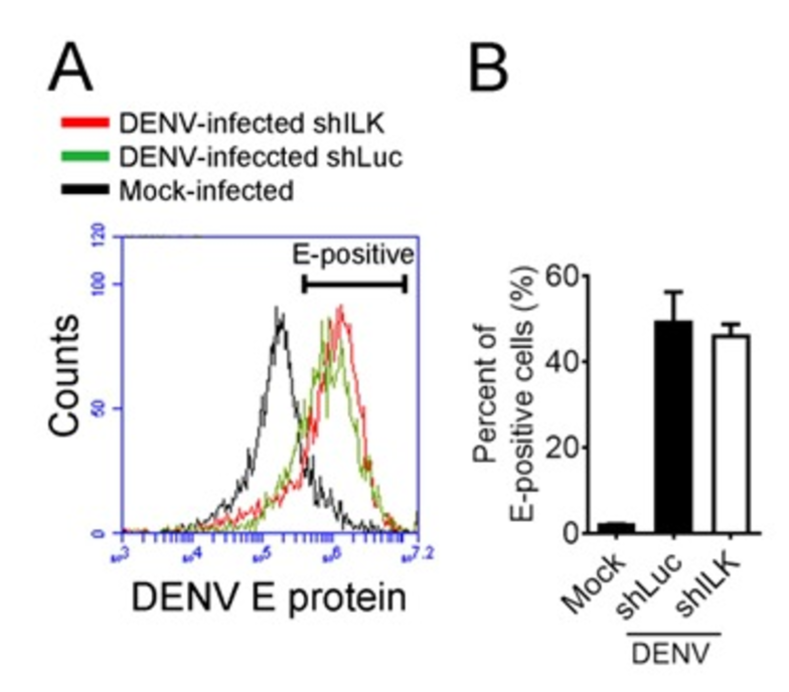

Supplement: S2 Fig — The control (shLuc) or ILK knockdown (shILK) A549 cells were infected with DENV at MOI of 25 at 4°C for 2 hours, washed, and stained with anti-envelope (E) antibody. (A) The representative histograms of mock-infected and DENV-infected control and ILK knockdown cells are shown. (B) The percentages of E-positive cells are shown. Data represent means + SD (error bars). (TIF) [file ppat.1011241.s003.tif]

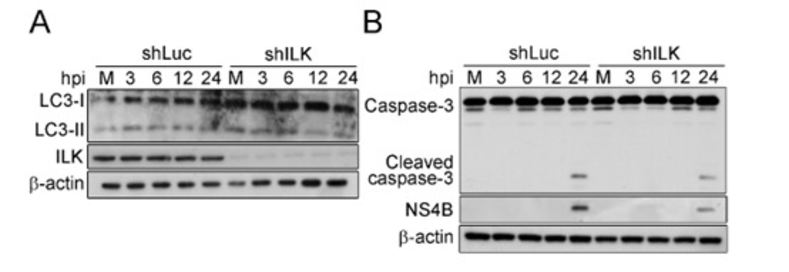

Supplement: S3 Fig — The representative western blots of indicated proteins in mock- (M) and DENV-infected control (shLuc) or ILK knockdown (shILK) A549 cells at indicated hours post-infection (hpi) are shown. (TIF) [file ppat.1011241.s004.tif]

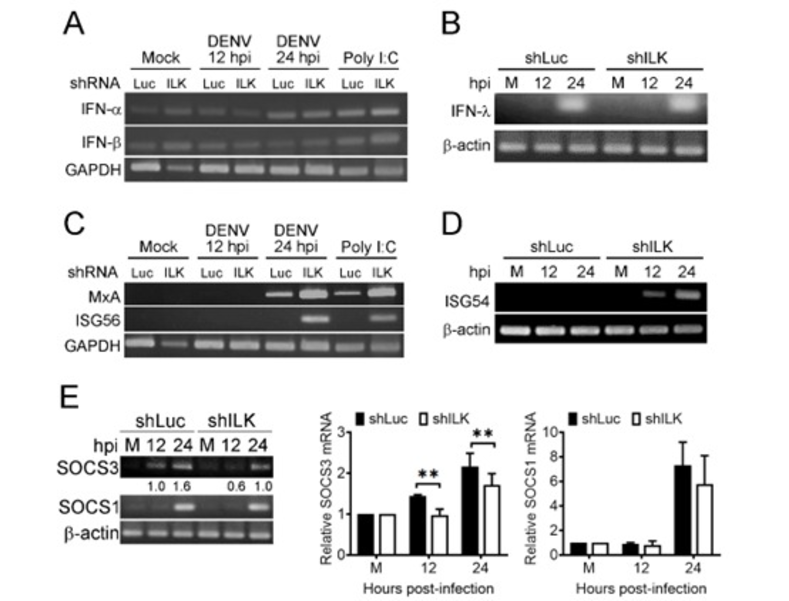

Supplement: S4 Fig — The control (shLuc) or ILK knockdown (shILK) A549 cells were mock- (M) or DENV-infected and collected at indicated hours post-infection (hpi) to determine the mRNA levels type I (α and β) IFN (A), type III (λ) IFN (B), ISGs, including MxA, ISG56, and ISG54 (C and D), SOCS1, and SOCS3 (E). The representative RT-PCR images of indicated genes are shown, and the ratios of SOCS3 to β-actin relative to control cells at 12 hpi are shown. The SOCS1 and SOCS3 mRNA levels determined by real-time PCR are shown (E, right panels). Cells treated with 10 μg/ml poly(I:C) for 24 hours served as positive controls. **p < 0.01. (TIF) [file ppat.1011241.s005.tif]

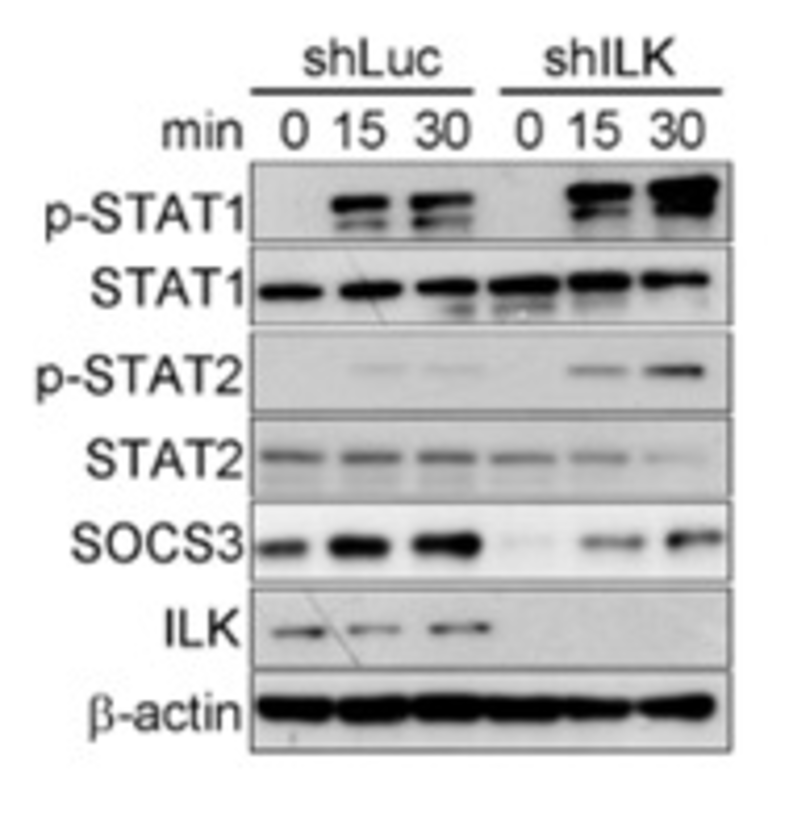

Supplement: S5 Fig — The representative western blots of indicated proteins in control (shLuc) or ILK knockdown (shILK) A549 cells treated with IFN-β (50 ng/ml) for indicated minutes (min) are shown. (TIF) [file ppat.1011241.s006.tif]

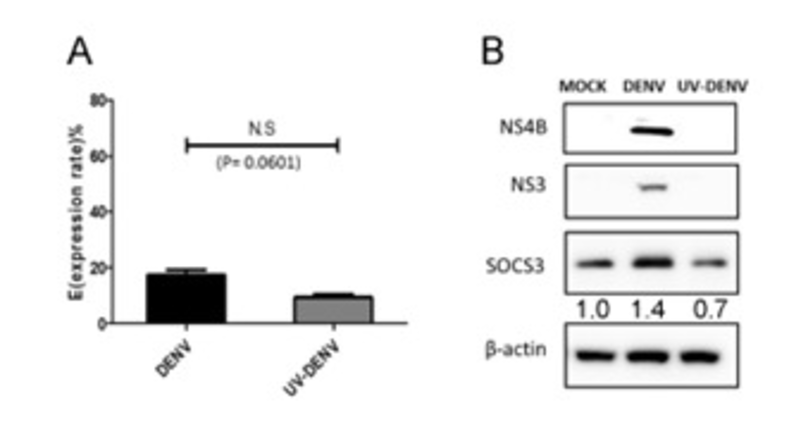

Supplement: S6 Fig — (A) A549 cells were infected with DENV or UV-inactivated DENV (UV-DENV) at MOI of 25 at 4°C for 2 hours, washed, and stained with anti-envelope (E) antibody. The percentages of E-positive cells analyzed by flow cytometry are shown. (B) The representative western blots of the indicated proteins in the A549 cells infected with DENV or UV-DENV for 24 hours are shown. The ratios of SOCS3 to β-actin relative to mock-infected cells are shown. Data represent means + SD (error bars) in panel A. N.S, not significant. (TIF) [file ppat.1011241.s007.tif]

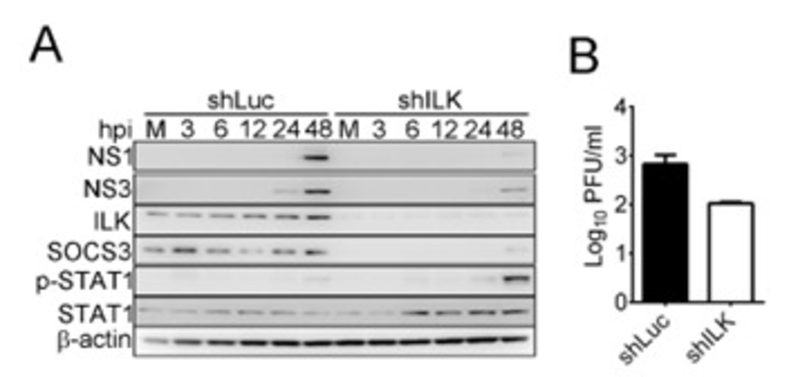

Supplement: S7 Fig — (A) The representative western blots of phosphorylated STAT1 (p-STAT1) and indicated proteins in control (shLuc) or ILK knockdown (shILK) cells infected with DENV at indicated hour post-infection (hpi) are shown. (B) The DENV yields in control and ILK knockdown cells at 24 hpi are shown. Data represent mean + SD (error bars). (TIF) [file ppat.1011241.s008.tif]

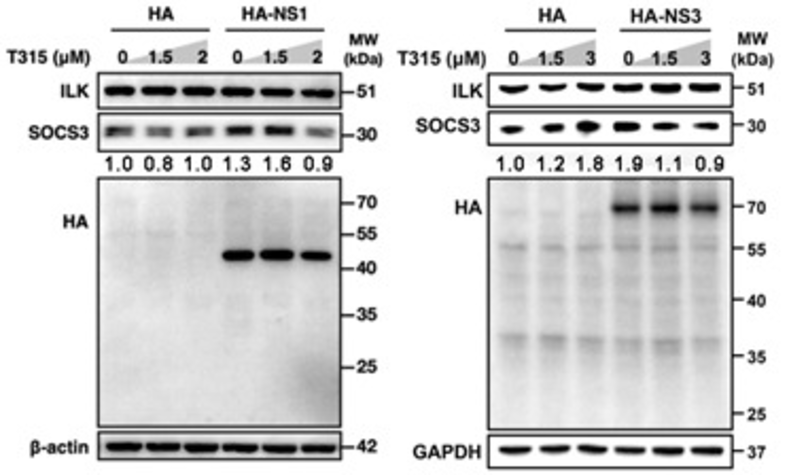

Supplement: S8 Fig — The representative western blots of indicated proteins in HA, HA-NS1 and HA-NS3 expressing A549 cells treated with OSU-T315 at indicated concentrations are shown. The ratios of SOCS3 to β-actin relative to HA-expressing cells without OSU-T315 treatment are shown. (TIF) [file ppat.1011241.s009.tif]

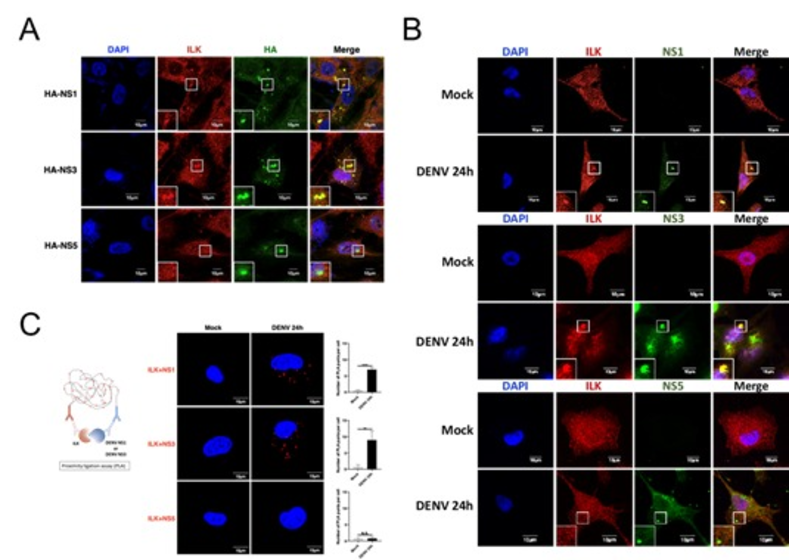

Supplement: S9 Fig — (A) The representative confocal images of HA-NS1, HA-NS3 and HA-NS5 expressing cells stained with ILK (green) or HA (red) are shown. (B) The representative confocal images of mock or DENV-infected cells stained with ILK (red) or NS proteins (green) are shown. (C) The representative images of proximity ligation assay in mock or DENV-infected cells are shown. (TIF) [file ppat.1011241.s010.tif]

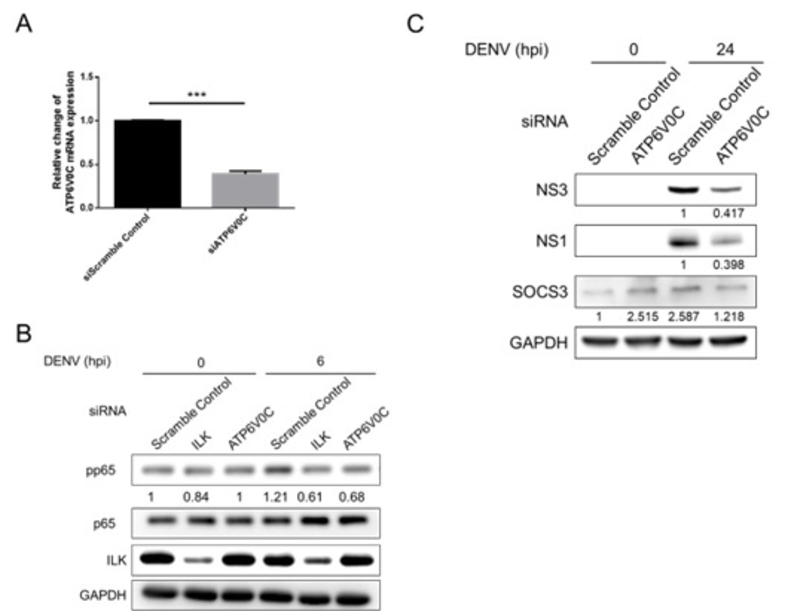

Supplement: S10 Fig — (A) ATP6V0C mRNA levels in A549 cells transfected with scramble or ATP6V0C-specific siRNA determined by qRT-PCR are shown. (B) The phosphorylated (p-) p65 and indicated proteins in the mock- or DENV-infected A549 cells transfected scramble control siRNA or siRNA targeting ILK or ATP6V0C at indicated hpi are shown. The ratios of phosphorylated to total p65 relative to mock-infected cells transfected with scramble siRNA at 0 hpi are shown. (C) The indicated A549 cells were infected with DENV (MOI of 2) for 0 and 24 hours. The NS1, NS3, SOCS3, and GAPDH protein expression in the indicated A549 cells are shown. The ratios of SOCS3 to GAPDH relative to mock-infected scramble control cells and the ratio of NS1 and NS3 to GAPDH relative to DENV-infected scramble control cells are shown. (TIF) [file ppat.1011241.s011.tif]
