# Supplementary material for: Negative regulation of type I interferon signaling by integrin-linked kinase permits dengue virus replication
Source: PLoS Pathog. 2023 Mar 17;19(3):e1011241. doi: 10.1371/journal.ppat.1011241 (PMC10057834; doi:10.1371/journal.ppat.1011241)
Supplement: Attachment — Submitted filename: Response letter_PPATHOGENS-D-22-01002.docx [file ppat.1011241.s012.docx]

Dr. Kasturi Haldar

The Editor-in-Chief

***PLOS Pathogens***

Dear Editor-in-Chief:

Thank you very much for processing our manuscript (#PPATHOGENS-D-22-01002). We have revised it according to the editors’ and reviewers’ comments and are re-submitting it for publication in ***PLOS Pathogens***. Please find our revised manuscript entitled “ Negative regulation of type I interferon signaling by integrin-linked kinase permits dengue virus replication” for your consideration for possible publication in ***PLOS Pathogens***. Two co-authors are added due to their contributions in this revised manuscript (Chien-Chou Chu on new Fig 5F, S1B Fig, Appendix I, II, III; Bo-Cheng Zhang on S1C and S1D Fig, Appendix IV, V). The electronic files of the revised manuscript containing text in marked copy, figures 1-7; and supplementary information containing 9 figures have been successfully transmitted through your online submission system. We appreciate the positive comments following the review of our original submission and have addressed each of the issues raised by the reviewers.

In particular, we would like to emphasize that the current study reveal that integrin-linked kinase (ILK) is a novel enhancing factor for DENV infection by suppressing type I interferon (IFN) responses. Mechanistically, ILK binds DENV NS1 and NS3, activates Akt and Erk, and induces NF-κB-driven suppressor of cytokine signaling 3 (SOCS3) expression. Elevated SOCS3 in DENV-infected cells inhibits phosphorylation of STAT1/2 and expression of interferon-stimulated genes (ISGs). Inhibiting ILK, Akt, or Erk activation abrogates SOCS3 expression. In DENV-infected mice, the treatment of an ILK inhibitor significantly reduces viral loads in the brains, disease severity, and mortality rate. Our findings highlight ILK is a potential therapeutic target against DENV infection. We believe that these findings would be of great interest to the readers of ***PLOS Pathogens*** and sincerely hope that our study will be considered for publication in your highly esteemed journal.

To enable a better understanding of our work, we have inserted a schematic diagram within the Figure section (please also see below)

Your kind assistance in evaluating this paper is highly appreciated.

Sincerely,

Chih-Peng Chang, Ph.D.,

Associate Professor

Department of Microbiology & Immunology, College of Medicine

National Cheng-Kung University

1 University Road, Tainan, Taiwan

Fax: 886-6-2082705

Tel: 886-6-2353535 Ext. 5643

e-mail: [cpchang@mail.ncku.edu.tw](mailto:cpchang@mail.ncku.edu.tw)

First, we would like to thank the editors and the reviewers for a very thorough and critical examination of our manuscript. We appreciate the helpful suggestions and comments which have been incorporated into our revised manuscript. Responses to specific comments are listed below.

**Reviewer #1**

1. Can the authors please comment on the fact that in figure 4 the authors show that there is upregulation of ISG54 and 56 in the absence of infection when SOCS3 is knocked down (4D lower panel, 0 h.p.i.). Does this impact the overall conclusion?

**Reply:** We thank the reviewer’s question. We did notice the upregulation of ISG 54 and 56 (**Fig 4B**) as well as a miner increase of p-STAT1 (**Fig 4C**) in mock-infected shSOCS3 A549 cells. This suggests that loss of SOCS3 increases intrinsic IFN-like signal and anti-viral gene expression in these cells, which we have no answer for it now. However, this signal leads to a reduction of viral replication in shSOCS3 cells (**Fig 4D**), indicating that SOCS3 inhibits IFN signal and acts as a pro-viral factor for DENV replication. We think that this argument is fit our conclusion.

2. In figure 5 and the overall conclusions of the paper, the authors imply that the NF-kB-mediated transcription of SOCS weights more on the infection that the pro-inflammatory genes. To support this the authors should measure virus growth (PFU/ml) in experiments (similar to) 5B, D and E.

**Reply:** As the reviewer suggested, we measured DENV yields in A549 cells treated with NF-κB, Erk, or Akt inhibitors at 24 hpi. **Fig 5F** shows at least 2-fold reductions in DENV yields in cells treated with the indicated inhibitors. We noted the results in **line 248**.

3. Can the results found in figure 6C be explained by the other immune modulatory functions already described for NS1 and NS3? The authors should include this in the discussion.

**Reply:** We thank the reviewer’s question. Previous studies revealed that overexpression of DENV NS1 and NS3 is able to induce IL-6 and IL-10 production, respectively (Chua *et al*. 2005. *Virus Res*. 112(1-2):85-94; Hurtado-Melgoza *et al*. 2016. *J Biomed Sci*. 23(1):85). Both IL-6 and IL-10 transduce a STAT3-dependent signal pathway, which has been shown to negatively regulate type-I IFN responses (Wang *et al*. 2011. *J Immunol*. 187(5):2578-85.). Thus, DENV NS1 and NS3 might also antagonize IFN signaling by activation of STAT3-mediated actions. We have added these descriptions to Discussion section (**line 341**).

**Reviewer #2**

1. The figure captions should be a bit more descriptive, such that a reader can get a good understanding of the experiments performed without referring to the main body of text. Figure 3 is a positive example. The caption for figure 3 makes it clear that all the images represent experiments performed with the A549 cell line. However, for figure 1, the A549 cell line is mentioned for some of the images but not the others. The figure 5 and figure S5 captions do not even mention what cell line is involved.

**Reply:** We appreciate the reviewer’s suggestions. We noted that “A549 cells were used in most experiments otherwise specified.” in the Materials and Methods (**line 398**) and have added the cell types used for experiments in revised figure legends.

2. The shRNA knockdown cell lines are used for most of the experiments in this study. The authors should include more details about the shRNA cell lines. Can the authors clarify the technical details of how the shILK knockdown cell lines are established? Was it used as a polyclonal pool, or were clonal cell lines selected after antibiotic selection?

**Reply:** We appreciate the reviewer’s suggestions. We included the methods for establishing shRNA cell lines in the Materials and Methods (**line 406**). All cell lines with shRNA were used as polyclonal pools.

3. For Huh-7, there are two knockdown cell lines: shILK-1 and shILK-2. Which was the shRNA sequence used to generate the knockdown cell lines for U937 and A549?

**Reply:** The shRNA targeting ILK 3’UTR was used to establish ILK knockdown in Huh-7 (shILK-1), U937, and A549 cells, and the shRNA targeting ILK CDS was used to generate Huh-7 shILK-2. To clarify the issue, we noted the information in the sections of Materials and Methods (**line 403**), Results (**line 134 and 168**), and Figure legends (**line 914**).

4. Can the authors elaborate on the shLuc that was used as a control shRNA? Does it target firefly luciferase? If so, it may not be an appropriate control for the replicon since it is expressing firefly luciferase. Wouldn’t the firefly luciferase RNA sequence in the replicon genome be targeted by the shLuc?

**Reply:** We thank the reviewer’s question and are sorry for this mislabeling issue. Actually, the control shRNA used in **Fig 2A** and **2B** is to target LacZ not luciferase. We have corrected this mislabeling in the revised **Fig 2**.

5. Authors should elaborate on some of the abbreviations used in the text and manuscript. For example, in figure 6, the abbreviation WCL should be defined in the caption as whole cell lysate.

**Reply:** We thank the reviewer’s suggestion. As suggested, we have carefully checked and added the abbreviation in the text and manuscript.

**Reviewer #3**

1. Fig 1A: levels of pAKT are increasing over time, both in control (shLuc) and ILK k.d. cells. The relative differences seems rather driven by overall lower protein amounts in kd cells (see ß-actin levels, lines 8-12 compared to 1-7). It would be important to confirm this observation in normalized WB, and show the corresponding cell viability of shILK-treated cells.

**Reply:** We thank the reviewer’s questions. We measured Akt phosphorylation level by calculating the ratio of phosphorylated to total Akt rather than phosphorylated Akt to actin. The results show that ILK knockdown did not affect A549 cell viability (**S1B Fig**) and reduced Akt phosphorylation in mock-infected and DENV-infected cells from 3 to 24 hpi about 1.5-fold (**line 134 to 137**).

2. Fig 1B. Are the concentrations at which the inhibitor reduces viral proteins levels cytotoxic? It would be important to show cell viability under the conditions used in this assay, as well as Akt phosphorylation levels upon inhibitor treatment to support its specificity.

**Reply:** We thank the reviewer’s question. The results were added in **S1 Fig**, showing that 1.5 to 3 μM OSU-T315 treatment reduced Akt phosphorylation without compromising cell viability (**S1C** and **S1D Fig**, respectively). We also noted the results in **line 142**.

3. Fig 1B vs Fig 2E: why the levels of NS4B or NS3 are mildly affected with i.e. 2.5 uM of OSU-T315, while in identical conditions in a different panel (Fig. 2E) appear almost completely abrogated?

**Reply:** We thank the reviewer’s questions. Viral proteins were almost completely abrogated in A549 cells treated with 3 μM OSU-T315 (**Fig 1B**) and in Huh7 cells treated with 2.5 μM OSU-T315 (**Fig 2E**). Hence, the concentration of OSU-T315 required to abrogated viral protein expression in A549 and Huh7 was not significantly different.

4. Fig 4B (lines 205-208): SOCS3 levels appear not upregulated by DENV infection when comparing i.e. 24h vs 0h post infection, while transcripts levels are comparable in both shLuc and shILK cells. Importantly shILK-mediated silencing does not significantly affect RNA levels of either SOCS1 or 3 in the agarose gel presented (Fig 4A). It would be important to see more quantitative methods to assess its levels rather than DNA electrophoresis (i.e. qRT-PCR). Similar concerns apply to S6Fig (“SOCS3 expression was abrogated”), which should support dependencies of these effects on active RNA replication (however differences in SOCS3 expression appear negligible).

**Reply:** We noted the SOCS3 to β-actin ratio in **S4E Fig** (original Fig 4A) and SOCS1 or SOCS3 to β-actin ratio in **Fig 4A** (original Fig 4B) and **S6**. Taken together, these results show that DENV increased SOCS3, but not SOCS1, mRNA and protein expression from 3 to 24 hpi, and ILK knockdown decreased SOCS3 expression in both mock- and DENV-infected cells. We modified the results section to clarify our point (**line 211 to 221**).

5. The mechanistic link of ILK to SOCS3 activity via NfKB axis mostly rely on virus-infections experiments in which viral replication is inhibited by silencing or drug treatment (see Lines 214-215; Fig. 4; Fig 5 D, E, etc..). However, to conclude that ILK is directly responsible for DENV-induced modulation of SOCS3 expression, it would be important to include controls in which similar reduction in virus replication is achieved (i.e. silencing of an unrelated host-depency factor such as ATP6V0C subunits).

Alternatively, is not possible to rule out pleiotropic effects resulting from lower replication of DENV (upon inhibition of ILK activity or any other host-dependency factor) which would equally result in reduced SOCS3 levels. Similarly, any other conclusion related to the interdependency of ILK and downstream IFN or NFkB pathway might be due to pleiotropic effects deriving from reduced viral replication (i.e. shILK reduces DENV replication, therefore is not surprising to observe slightly lower pERK levels in these conditions) (Fig. 5C).

Notably, experiments which are independent from active viral replication (i.e. ectopic expression of NS1 or NS3 followed by treatment with t315 inhibitor) display negligible effects on SOCS3 abundance upon inhibitor treatment (and protein expression as well)). i.e. Fig. S8.

**Reply:** We thank the reviewer for the comment. The mock-infected cells were collected and noted as 0 hpi in the original manuscript. To be more specific, we noted it as mock-infected (M) in the revised manuscripts and added the details in the Material and Methods (**line 434**). Hence, the results show that in mock-infected cells, ILK knockdown abolished SOCS3 expression (**Fig 4A** and **S7**) and signaling required for SOCS3 expression, including phosphorylated Akt and p65 (**Fig 1 and 5**), indicating that ILK is required for SOCS3 expression independent of DENV infection (**line 222 to 226**).

Moreover, DENV replication is required for elevating SOCS3 expression (**S6 Fig**), implying that viral proteins hijack ILK-regulated signaling to increase SOCS3 expression. Indeed, we show that DENV NS1 and NS3 interacted with ILK and hijacked the pathway to increase SOCS3 expression (**Fig 6**). Accordingly, genetic or pharmacologically blockage of ILK and its signaling diminished DENV-induced SOCS3 expression (**Fig 4** and **5**, respectively). For **S8 Fig**, we noted the ratios of SOCS3 to β-actin in **S8 Fig**, showing that ectopic NS1 and NS3 expression stimulated SOCS3 expression in A549 cells, and OSU-T315 decreased the NS1- and NS3-induced SOCS3 expression by 40 to 50%.

Knockdown of ATP6V0C, a DENV host-dependency protein, via siRNA diminished ATP6V0C mRNA by 50% (0.48 ± 0.01 in cells with siATP6V0C compared scramble siRNA) but increased SOCS3 expression without DENV infection. Of note, knockdown of ATP6V0C slightly reduced DENV NS1 mRNA levels at 24 hpi by 32 % and showed comparable SOCS3 expression with control cells (**Appendix I**).

**Appendix I. SOCS3 expression in A549 cells with ATP6V0C knockdown.** (A) The mRNA levels of ATP6V0C in A549 cells transfected with scramble or ATP6V0C-specific siRNA determined by qRT-PCR are shown. (B) The indicated A549 cells were infected with DENV (MOI of 2) for 0, 12, and 24 hours. The mRNA levels of NS1 determined by qRT-PCR are shown. (C) The indicated A549 cells were infected without (Mock) or with DENV (DV, MOI of 2) for 24 hours. The ratios of SOCS3 to GAPDH relative to mock-infected cells transfected with scramble siRNA are shown.

6. While the interaction between NS1 and NS3 with ILK is convincingly supported by co-IP-WB and PLA experiments, the increased recruitment of Akt to ILK upon NS1 or NS3 expression is not supported by the data presented in the manuscript. In fact, despite NS1 and NS3 overexpression significantly reduce ISG56 levels (Fig. 6C), AKT-ILK stochiometry appear unaffected by NS1 or NS3 expression (see Akt levels comparable in all lanes, Fig.6G), therefore hampering the conclusion that NS1 and NS3 interact with ILK to attenuate type1 IFN responses. Similarly, it would be important to overexpress an unrelated ER soluble protein to ensure that increased pAkt and NFkB levels are due to NS1 and NS3 and not deriving from overexpression artefacts (NS5 is expressed in the nucleus and cytoplasm and might actually counteract those pathways in directly) (Fig 6A)

**Reply:** We added the ratios of Akt co-immunoprecipitated with ILK to ILK precipitated by anti-Flag antibody in **Fig 6G**. The ratio is 1.0, 2.6, and 4.8 in ILK overexpressing cells transfected with empty vector, NS1, and NS3, respectively. The results support our conclusion that NS1 and NS3 facilitate Akt-ILK interaction. Furthermore, the p65 phosphorylation levels were higher in A549 cells with ectopically expressed NS1 and NS3 than SEC61B, an unrelated ER soluble protein (**Appendix II**), indicating that p65 phosphorylation is not driven by overexpression artifacts.

**Appendix II. Ectopic expression of NS1 and NS3, but not SEC61B, increases p65 phosphorylation.** Western blots of phosphorylated and indicated proteins in A549 cells without (Mock) or with ectopically expressed NS1, NS3, or SEC61B are shown. The phosphorylated to total p65 ratios relative to mock cells are shown.

7. Lines 221-222 (Fig 4E): “Furthermore, SOCS3 knockdown enhanced p-STAT1/2 and suppressed NS1 and NS4B expression in infected cells (Fig 4E)”. This blot actually shows upregulation of pSTAT1 levels in both shLuc and shSOCS3 silenced cells, suggesting that hyperphosphorylation of STAT1 is dependent on viral infection but SOCS3-independent.

**Reply:** We thank the reviewer’s comment. As we noted in **Fig 4C**, SOCS3 knockdown increased STAT1 phosphorylation (**lane 1 and 3**) as well as ISG54 and ISG56 (**Fig. 4B**) in cells without DENV infection. This suggests that loss of SOCS3 increases intrinsic IFN-like signal and anti-viral gene expression in these cells. DENV infection stimulates STAT1 phosphorylation in control and SOCS3 knockdown cells. Indeed, the STAT1 phosphorylation was not further enhanced by SOCS3 knockdown during DENV infection. However, SOCS3 knockdown resulted in STAT1 activation and ISG expression before DENV infection. The elevated antiviral status in SOCS3 knockdown cells restricted DENV replication. Hence, STAT1 phosphorylation did not further enhance by SOCS3 knockdown in DENV infected cells. We have changed the descriptions in Result section (**line 213 to 217**).

8. Lines 230-232 (Fig 5A) “Our data showed that DENV infection increased the phosphorylation of p65 on Ser536 (p-p65) in control cells while ILK knockdown abrogated this p65 phosphorylation”. In the blot presented, p-p65 levels appear comparable (left top blot; pp65 levels i.e. at 3h and 25h.p.i., shLuc treated cells). Is the hyperphosphorylation of p65 dependent on ILK? How pp65 levels vary upon silencing of any known DENV host-dependency factors (i.e. under reduced viral replication, independently from ILK levels?)

**Reply:** We noted the phosphorylated to total p65 in **Fig 5A**, showing that DENV infection increased p65 phosphorylation from 3 to 24 hpi, and ILK knockdown abrogated p65 phosphorylation in mock- and DENV-infected cells.

To understand whether p65 phosphorylation depends on ILK, we monitored it in ATP6V0C knockdown cells (Appendix III). Knockdown of ATP6V0C did not affect ILK expression in mock or DENV-infected cells. The reduction of p-p65 was observed in both ILK and ATP6V0C knockdown DENV-infected cells compared with control cells. This suggests that the hyperphosphorylation of p65 in DENV-infected cells can be regulated by ILK and ATP6V0C.

**Appendix III. The phosphorylated (p-) p65 expression in A549 cells with ILK or ATP6V0C knockdown.** The phosphorylated (p-) p65 and indicated proteins in the mock- or DENV-infected A549 cells transfected scramble control siRNA or siRNA targeting ILK or ATP6V0C at indicated hours post-infection (hpi) are shown. The ratios of phosphorylated to total p65 relative to mock-infected cells transfected with scramble siRNA at 0 hpi are shown.

**Part III – Minor Issues: Editorial and Data Presentation Modifications**
**Reviewer #1**

1. line 237 reads "treatment of the ERK inhibitor...", did the authors meant "with ERK inhibitor"?

**Reply:** We thank reviewer’s question. We did mean treatment “with ERK inhibitor”. The description has been corrected **(line 244)**.

2. please align the blot boxes. This is particularly an issue with Figure 5A.

**Reply:** We aligned the blot boxes.

**Reviewer #2**:

1. Figure S1 and S7. It may be a bit redundant, but the authors can consider giving S1 its own set of WB images showing that ILK expression is knocked down in the shILK U937 cell line. The knockdown of ILK is also shown in S7 but this is much later in the manuscript.

**Reply:** We moved the S1 to **S7B Fig** and noted the results in **line 226 to 230**.

2. Figure 1: 1A shows there is successful knockdown of ILK. However, authors may consider adding an additional untransduced control cell line for the western blot. The additional western blot could be included in the SI.

**Reply:** We thank the reviewer’s suggestion and included the western blots of DENV2-infected, untransduced A549 cells in **S1A Fig** and Results section (**line 131**).

3. Figure 1A: The authors should include a column plot that shows the values of AKT and AKT-P473 normalised to actin.

**Reply:** We added the phosphorylated to total Akt ratio in the Fig 1A and noted that all western blots are representative images from 2 independent experiments (**line 450**).

4. Figure 2: Why was Huh-7 used for the replicon experiments? Wouldn’t the A549 be a better cell line compared to the Huh-7 cell line, especially since the Huh-7 cell line may be deficient in some of the interferon pathway components? E.g Huh-7 deficient in MDA5? Or is the A549 cell line non-permissive for the replicon?

**Reply:** We used Huh7 cells, which are competent for RIG-I and MDA5-mediated signaling (Nasirudeen *et al*, 2011. *PLoS. Negl. Trop. Dis.* 5:e926; Loo *et al*, 2008. *J. Virol.* 82:335-345.), instead of the Huh7.5 cells, which are defected in RIG-I-mediated signaling (Sumpter *et al*, 2005. *J. Virol.* 79:2689-2699). The Huh7 cells carrying DENV2 replicon was established previously (Su *et al*, 2016. *J. Virol.* 90:4308-4319) and used assess the role of ILK in DENV replication after binding and entry. Additionally, the Huh7 cells are used to show that ILK knockdown reduces DENV infection in a manner not specific to a particular cell type.

5. Figure S2: binding and entry. The authors used an interesting approach to investigate virus binding to the host cell. Instead of quantifying viral RNA using qRT-PCR, the authors performed immunostaining using anti-Env antibodies and analysed the immunostaining using flow cytometry. Can the authors clarify if this method is just as sensitive as the qRT-PCR method? Can the authors also clarify how they defined the gating for S2B? For example, given the gating they used to define Env positive cells, what is the background positive rate from the mock-infected cells? The authors can consider including the mock-infected cells in the graph for S2B.

**Reply:** We performed binding assay via flow cytometry as previous described (Chen *et al*. 2017. *Antiviral Re*s. 142:158-168). The gating of E-positive cells was shown in **Appendix IV** (upper panel). The percentage of E-positive cells in mock group around 2.4 % and had been added in **S2B Fig**. We also performed the DENV binding via quantifying viral RNA using qRT-PCR (**Appendix IV**, lower panel). At MOI of 25, the virus RNA on these cells was increased by 15-20-fold compared with mock groups. Similarly, E-positive cells was increased by around 20-fold compared with mock groups by flow cytometry at the same MOI (25). We think that immunostaining using anti-E antibodies via flow cytometry is just as sensitive as the qRT-PCR method to detect DENV binding.

**Appendix IV. DENV binding on cell surface analyzed by flow cytometry and real-time PCR.** A549 cells were infected without (Mock) or with DENV at MOI of 25 (upper panel) or indicated MOI (lower panel) for 2 hours at 4^o^C, washed to remove the unbound virus, and subjected to flow cytometry (upper panel) or qRT-PCR (lower panel). The E-positive cells was gated from P2 region. Data represents mean + SD (error bars) in the bar graph. **p* < 0.05, ****p* < 0.001.

6. Line 174 to 175: The authors reference some papers and state that “DENV has been known to induce the formation of autophagosomes as replication compartments in cells” Unfortunately, this is an older hypothesis that has been superseded. The DENV replication complex is derived from the ER membrane, not autophagosomes. More recent studies show that induction of autophagy seems to be required for hijacking host lipid metabolism (among other things). The authors should revise this sentence.

**Reply:** We have revised the sentence (**line 178**) and included the reference showing that DENV induces lipophagy as reviewer’s suggestion.

7. Figure 6 and for the main text of the manuscript related to figure 6: the authors should elaborate on the HA and flag tagged constructs. Are the HA and flag tags at the N-terminus or the c-terminus? A diagram of the design of the HA and flag tagged constructs might help, especially if the authors can show the location of the ANK repeats, PH domain, and kinase domain in relation to the full length, 1-170, and 171-452 constructs.

**Reply:** We thank the reviewer for the suggestion. We added the details of HA- or Flag-tagged plasmids in the Materials and Methods (**line 412**).

8. Figure 7 and discussion related to TNF-a and IL6. Is the decrease in TNF-a and IL6 levels an off target effect of OSU-T315, or is it caused by the lower viral load in the mice?

**Reply:** We thank reviewer’s question. In Figure 7, we observed the comparable levels of both TNF-a and IL-6 in mock-infected mice those treated with or without OSU-T315. This indicates that OSU-T315 shows no effects on production of these two cytokines. Since DENV infection caused elevated levels of TNF-a and IL-6 in mice (**Fig 7E**), we think that the reduction of TNF-a and IL-6 in OSU-T315 treated mice is caused by lower viral load (**Fig 7C**).

9. Figure 7D: how were the mouse brain sections prepare after embedding? E.g how were they sectioned? Do the authors have the brightfield images? The nuclei count looks quite different between the sections for the different treatment groups.

**Reply:** We specified the methods to prepare mouse brain sections in **line 454 to 458**. The images are taken from the cerebral cortex where DENV was injected, and the information was added in the legends of Fig 7D (**line 989**). The nuclear count was increased in brain sections after DENV infection and was reduced after OSU-T315 treatment. The results imply that immune cells are infiltrated into infected mouse brains and that OSU-T315 treatment reduced it.

**Reviewer #3**

1. Fig5D: the inhibitor (U0126) is used at relatively high concentrations (up to 100uM). Are there any data on the cell viability under these conditions?

**Reply:** We thank reviewer’s question. The cell viabilities of these U0126-treated cells (from 5 to 100 uM) were over 90 % (**Appendix V**).

**Appendix V. The viability of A549 cells treated with U0126.** The viability of A549 cells treated with indicated concentration of U0126 or 0.05% Triton X-100 for 24 hours are shown. Data represent mean + SD (error bars).

2. Throughout the manuscript the authors use the wording “ILK enhances DENV infection”, however it might as well act as an host-dependency factor; it would be more appropriate to change the wording.

**Reply:** We thank the reviewer’s suggestion and revised our description in the manuscript.

3. Line 163:“ILK fails to affect virus entry”-> ILK does not play a role in virus entry

**Reply:** We modified the original line 163 (now **line 165**) as “ILK does not play a role in virus binding.”

4. Fig 2D: please express the % of cell viability as fold-of-control rather than 0.05% TritonX. When displayed as fold of TritonX is difficult to derive whether they were cytotoxic compared to non-treated cells.

**Reply:** We thank the reviewer’s suggestion. We revised Fig 2D and showed that OSU-T315 treatment did not compromised the viability of Huh7 cells.
