# Supplementary material for: Negative regulation of type I interferon signaling by integrin-linked kinase permits dengue virus replication
Source: PLoS Pathog. 2023 Mar 17;19(3):e1011241. doi: 10.1371/journal.ppat.1011241 (PMC10057834; doi:10.1371/journal.ppat.1011241)
Supplement: Attachment — Submitted filename: P to P-R1.docx [file ppat.1011241.s013.docx]

We would like to thank the editors and the reviewers for a very thorough and critical examination of our manuscript. We appreciate the helpful suggestions and comments which have been incorporated into our revised manuscript. Responses to specific comments are listed below.

1. The manuscript is now improved and authors have included some experiments which partially strengthen their conclusions, namely the finding that NS1 and/or NS3 bind to ILK; and that SOCS3 expression is decreased upon ILK silencing. Furthermore, they have now included some data supporting the lack of cytotoxic effects by some of the compounds or shRNA used. However, this reviewer feels that evidence supporting the modulation of SOCS3 abundance by viral infection by WB or RT-PCR presented (Fig.4A and B respectively; addressed in Point 4 of rebuttal) is still quantitatively inconclusive and would require a quantitative assay (i.e. qRT-PCR; n=3), or a substantial re-phrasing.

**Reply:** We thank the reviewer's comments. We have performed qRT-PCR to determine the mRNA levels of SOCS1 and SOCS3 in mock- and DENV-infected control and ILK knockdown A549 cells and added the results in the supplementary information (**S4E Fig**). DENV infection increased SOCS3 expression in both control and ILK knockdown cells. SOC3 mRNA levels were 1.4- and 1.3-fold higher in control cells than in ILK knockdown cells at 12 and 24 hours post-infection (hpi), respectively.

2. The additional experiments performed to support the lack of pleiotropic effects underlying SOCS3 reduction presented in Appendix 1 (addressed in Point 5 of the rebuttal) are not conclusive. In this experiment the authors aimed to show that k.d. of an unrelated host protein, would not result in pleiotropic reduction of SOCS3 levels due to decreased viral replication (rather than directly to ILK abundance). However, upon k.d. of ATP6V0C they observe a very mild reduction of DENV replication and only at 72h.p.i. using a quantitative approach (32% reduction in viral replication by qRT-PCR) while conclude that no effects can be observed on SOCS3 expression by WB (performed on lysates at 24.h.p.i.; where no reduction in viral replication can be observed by qRT-PCR). Indeed, by WB one would probably not be able to measure even reduced viral antigen under such mildly reduced viral replication condition. Could the authors show the levels of any viral antigen in this experiment?

**Reply:** We agreed with reviewer’s concern that knockdown of ATP6V0C showed only mild reduction of DENV replication in our revious results, which might be caused by low knockdown efficiency (less than 50%). Thus, we have optimized the experimental condition to increase the ATP6V0C knockdown efficiency to more than 60% (0.38 ± 0.01 in cells with siATP6V0C compared scramble siRNA; **Appendix I and S10 Fig**). After DENV infection, we determined viral proteins (NS1 and NS3) and SOCS3 expression at 24 hpi. Consistent with our previous results (point 5 in the previous rebuttal), ATP6V0C knockdown increased SOCS3 expression in mock-infected cells. After DENV infection, ATP6V0C knockdown reduced NS1 and NS3 protein levels by 60% and decreased SOCS3 expression by 2-fold. The results might be partly due to reduced NS1 and NS3 interaction with ILK, which supports our conclusion (**Fig. 7F**). However, we could not rule out that DENV infection might activate other signalings to increase SOCS3 expression, as the reviewer suggested. We have added these data in new **S10 Fig** and modified the Discussion section to address the issue (line 330-336).

**Appendix I**. ATP6V0C mRNA levels in A549 cells transfected with scramble or ATP6V0C-specific siRNA determined by qRT-PCR are shown (left panel). The indicated A549 cells were infected with DENV (MOI of 2) for 0 and 24 hours. The NS1, NS3, SOCS3, and GAPDH protein expression in the indicated A549 cells are shown (right panel). The ratios of SOCS3 to GAPDH relative to mock-infected scramble control cells and the ratio of NS1 and NS3 to GAPDH relative to DENV-infected scramble control cells are shown.

3. Along these lines, still no further evidence supporting a causal connection between ILK->pp65 is provided (Point 8 of the rebuttal). A variation of 0.6 fold (from 2.0 to 2.6, according to densitometry presented in Fig. 5A; shLuc lanes) in pp65 levels by WB, does not corroborate the notion that ILK is involved in p65 phosphorylation.
Coherently and importantly, the new experiments performed by the authors (Appendix III) clearly show that k.d. of ATP6V0C (a totally unrelated host protein, which also reduces viral replication) reduce levels of pp65 to a very similar levels that ILK k.d. Altogether, the evidence presented actually consolidate the notion that the reduction of pp65 is unrelated to ILK or SOCS3 levels, but rather a pleiotropic effect of reduced viral replication (conversely the authors conclude that also ATP6V0C – i.e. inhibition of endocytosis - is involved in pp65 regulation).

**Reply:** We thank the reviewer’s comments. According to the data in Fig. 5A, the p-p65 level was reduced by 90% in mock-infected ILK knockdown cells (lane 6) compared to control cells (lane 1). The similar significant reduction was clearly observed in ILK knowndown cells compared with control cells at 3 , 12 and 24 hpi after DENV infection. These data indicate that ILK is involved in p65 phosphorylation with and without DENV infection.

As we responded in point 2, ATP6V0C knockdown reduced p-p65 might partly result from decreased NS1/NS3 and ILK interaction and other signalings that have not been identified. For example, a study demonstrated that inhibition of endocytosis down-modulates IL-1-induced NF-κB-dependent gene expression (Cell Signal. 2013 Jan;25(1):214-28.). However, we could not rule out that DENV infection might activate other signalings to increase p-p65 as the reviewer suggested. We have added the data in new **S10 Fig** and addressed the issue in the Discussion section (line 330-336).
