# Supplementary material for: Negative regulation of type I interferon signaling by integrin-linked kinase permits dengue virus replication
Source: PLoS Pathog. 2023 Mar 17;19(3):e1011241. doi: 10.1371/journal.ppat.1011241 (PMC10057834; doi:10.1371/journal.ppat.1011241)
Supplement: Attachment — Submitted filename: P to P-R2.docx [file ppat.1011241.s014.docx]

We would like to thank the editors and the reviewers for a very thorough and critical examination of our manuscript. We appreciate the helpful suggestions and comments which have been incorporated into our revised manuscript. Responses to specific comments are listed below.

Thank you for addressing many reviewers comments, including the quantitative qPCR probing SOCS3 levels after infection in ILK knockdown cells. Please address why and how the small change (1.4-1.3X) in SOCS3 levels would be the driving factor behind the phenotype (in either text or via experiment)?

**Reply:** We thank reviewer's question. We did find that SOCS3 mRNA levels were only 1.4- to 1.3-fold higher in control cells than in ILK knockdown cells after DENV infection (**S4E Fig**). However, the protein levels of SOCS3 were dramatically reduced in ILK knockdown cells with or without DENV infection compared to control cells (**Fig 4A**). A recent study revealed that ILK is able to increase oncogenic protein synthesis by positively regulating translation via AKT/mTOR/eIF4E pathways (Int J Mol Sci. 2021 Apr 1;22(7):3682.). According to these observations, we suggest that ILK might upregulate both SOCS3 RNA expression and translation as well to support DENV infection. We have addressed the issue in the Discussion section (line 336-344).
